# Loss of lactate dehydrogenase A disrupts R-loop homeostasis and induces DNA damage in breast cancer cells

**DOI:** 10.3389/fgene.2026.1942597

**Published:** 2026-09-14

**Authors:** Shangzhen Li, Cheng Xu, Shuling Chen, Guoping Yin, Dawei Yang

**Affiliations:** 1 Nanjing Key Laboratory of Hepatology, The Second Hospital of Nanjing, Affiliated to Nanjing University of Chinese Medicine, Nanjing, China; 2 Department of Pharmacy, The Second Hospital of Nanjing, Affiliated to Nanjing University of Chinese Medicine, Nanjing, China; 3 Department of Anesthesiology, The Second Hospital of Nanjing, Affiliated to Nanjing University of Chinese Medicine, Nanjing, China

**Keywords:** breast cancer, Cut&Tag, genomic stability, lactate dehydrogenase A, R-loop, transcription

## Abstract

**Introduction:**

R-loops, three-stranded nucleic acid structures composed of an RNA-DNA hybrid and displaced single-stranded DNA, are tightly regulated by single-stranded DNA-binding proteins. However, the role of lactate dehydrogenase A (LDHA), an atypical DNA-binding protein and key glycolytic enzyme, in R-loop regulation remains unclear.

**Methods:**

Cleavage Under Targets and Tagmentation sequencing (CUT&Tag-seq) was utilized to map R-loop and LDHA genomic landscapes in wild-type (WT) and LDHA knockout (KO) 4T1 cells. RNA-seq was employed for transcriptomic profiling. Immunofluorescence was used to evaluate the co-localization of LDHA and R-loops. Western blotting was performed to assess the protein levels of LDHA, RNase H1, and γ-H2AX.

**Results:**

LDHA knockout disrupted R-loop homeostasis, resulting in altered S9.6-enriched peaks, with 199 peaks increased and 81 decreased in 4T1 cells. Exogenous lactate failed to rescue these R-loop perturbations, suggesting that LDHA regulates R-loops through a mechanism beyond its metabolic activity. Genomic analysis revealed partial overlap between LDHA-binding sites and R-loop-prone regions, indicating a potential direct role of LDHA in R-loop formation. Transcriptomic profiling showed widespread gene expression changes following LDHA loss, although only four genes were associated with both LDHA binding and R-loop peaks. Importantly, LDHA knockout induced accumulation of R-loop foci and DNA damage, both of which were reversed by RNase H1 expression.

**Discussion:**

These findings identify LDHA as a novel regulator of R-loops and suggest that it may influence R-loop homeostasis through direct DNA binding rather than solely through its enzymatic role in glycolysis. Further studies are needed to elucidate the molecular mechanism underlying LDHA-mediated R-loop regulation.

## 1 Introduction

R-loops are three-stranded nucleic acid structures composed of an RNA:DNA hybrid and a displaced single-stranded DNA (ssDNA) (Camino et al., 2023; Petermann et al., 2022). They predominantly form co-transcriptionally when a nascent RNA molecule hybridizes back to the DNA template strand (Lim et al., 2023). While R-loops play physiological roles in processes such as transcription regulation (Hwang et al., 2025; McCann et al., 2023), DNA repair (Davó-Martínez et al., 2023; Girasol et al., 2023a), chromatin organization (Herrera-Moyano et al., 2014), telomere maintenance (Xu et al., 2024), and immune evasion in certain organisms (Girasol et al., 2023b), their aberrant accumulation or persistence is a significant source of genomic instability (Camino et al., 2023; Ren et al., 2024; Herrera-Moyano et al., 2014). Unresolved R-loops can impede DNA replication and transcription, leading to replication stress, DNA damage, and transcription-replication conflicts, which contribute to mutagenesis and diseases (Hamperl et al., 2017; Marabitti et al., 2019; Parajuli et al., 2017). Consequently, cells have evolved intricate mechanisms to tightly regulate R-loop levels, balancing their formation and resolution. One key class of factors regulating R-loops is Single-Strand Binding proteins (SSBs).

SSBs are a class of proteins that bind to single-stranded DNA. Since R-loops possess a ssDNA structure, SSBs tend to bind to these structures. Several studies have demonstrated that SSBs can influence R-loop dynamics by either promoting or hindering their formation. For example, the Organellar Single-stranded DNA Binding Protein 1 (OSB1) in Arabidopsis suppresses R-loop formation, thereby maintaining mitochondrial DNA integrity and supporting normal embryogenesis (Cheng et al., 2021). In *E. coli*, SSB facilitates R-loop removal by localizing RNase H1 to replication forks (Wolak et al., 2020). Similarly, in human hematopoietic stem cells, the loss of SSBs (SSB1/SSB2) leads to the accumulation of R-loops and subsequent genomic instability, implying a role in R-loop resolution or prevention (Shi et al., 2017). Conversely, some SSBs are implicated in promoting the formation or persistence of R-loops. The human mitochondrial SSB stimulates R-loop formation, which is a necessary intermediate for origin-specific mtDNA replication initiation following processing by RNase H1 (Posse et al., 2019). Furthermore, the Arabidopsis SSB AtNDX promotes the persistence of an R-loop involved in regulating the expression of a long noncoding RNA (Sun et al., 2013). Therefore, the impact of SSBs on R-loops is not uniform; it varies depending on the specific SSB, the cellular context, and the organism.

LDHA is an atypical SSB, traditionally known for its role in glycolysis. LDHA catalyzes the reduction of pyruvate to lactate, a reaction central to anaerobic glycolysis (Fantin et al., 2006). Under normal physiological conditions, this function helps regenerate NAD^+^ from NADH, ensuring the continuation of glycolysis in the absence of oxygen (Sharma et al., 2022). This classical metabolic function is crucial for energy production, particularly in conditions where oxidative phosphorylation is limited, such as in rapidly proliferating cells or in hypoxic tumor environments (Sharma et al., 2022). In addition to this canonical function, LDHA has also been reported to have some non-classical functions, such as catalyzing the production of L-2HG from α-KG under acidic conditions (Intlekofer et al., 2017), promoting the activation of Rac1-GTP through direct protein-interaction (Liu et al., 2022), and, as related to this manuscript, its single-stranded DNA binding function to regulate transcription and DNA replication (Fiume et al., 2013; Grosse et al., 1986). However, as a highly regarded metabolic enzyme and non-classical SSB, its influence on R-loops is almost unknown. Thus, we constructed LDHA-Knockout (LDHA-KO) 4T1 cells using crispr-cas9, and compared the genome-wide abundance of R-loops in wild-type cells with LDHA-KO cells through multi-omics means.

In the present study, we demonstrated that LDHA knockout promotes R-loop accumulation, that LDHA DNA-binding sites partially overlap with R-loop-associated sequences, and that LDHA physically interacts with R-loop-like substrates. Meanwhile, although our assays did not establish a role for R-loops in LDHA-mediated transcriptional regulation, we observed that R-loop degradation by RNase H1 attenuated the DNA damage induced by LDHA knockout. This suggests that R-loop accumulation contributes to the genomic instability caused by LDHA depletion. Ultimately, these findings advance our understanding of the non-canonical functions of LDHA and provide new insights into the regulatory mechanisms governing R-loop homeostasis.

## 2 Methods

### 2.1 Cells

The murine breast cancer cell line 4T1 was procured from the Cell Bank of the Chinese Academy of Sciences (Shanghai, China) and cultured in RPMI-1640 medium (KeyGEN BioTECH, Nanjing, China) supplemented with 10% heat-inactivated fetal bovine serum (FBS; YEASEN, Shanghai, China). All cell cultures were maintained in a humidified atmosphere containing 5% CO_2_ at 37 °C during their exponential growth phase.

### 2.2 Gene editing

LDHA in 4T1 cells was knocked out using a CRISPR Gene Knockout Kit (Cas9X, Suzhou, China). Homozygous knockout cells were then selected from monoclonal colonies, and the successful knockout was verified by Sanger sequencing and Western blotting.

gRNA-A1: CTT​AGC​ATG​AGA​CCG​TGC​GG TGG

gRNA-A2: CCA​GTG​CCC​TAG​CCT​TAC​GG AGG.

### 2.3 CUT&Tag-seq

The Hyperactive Universal CUT&Tag Assay Kit for Illumina Pro (Vazyme, Nanjing, China) was utilized for this assay according to the manufacturer’s instructions, with some modifications for R-loop and LDHA detection. Briefly, 10,000 viable cells were collected, permeabilized, and sequentially incubated with ConA beads Pro, primary antibody, secondary antibody, and pA/G-Tnp Pro. Subsequently, DNA was fragmented, extracted, and purified. Libraries (TruePrep Index Kit V4 for Illumina, Vazyme, Nanjing, China) were amplified and then sequenced using next-generation sequencing (NGS) on an Illumina platform (GENEWIZ, Suzhou, China). Raw sequencing data were analyzed *via* the Vazyme Cloud Analysis Platform against the mm10 mouse reference genome. Peaks were defined as significantly differentially enriched based on a Wald test *p*-value <0.05 and an |Mval| > 0.57.

For R-loop detection, a control group was established by incubating cells with 10 U of RNase H (YEASEN, Shanghai, China) in antibody buffer at room temperature for 30 min prior to the addition of the primary antibody. Both the control and experimental groups were then incubated with 2 µg of S9.6 primary antibody (Abcam, Boston, USA) at 4 °C overnight.

For LDHA detection, the control group was incubated with 2 µg of Control IgG (ABclonal, Wuhan, China), while the experimental group received 2 µg of LDHA primary antibody (Finetest, Wuhan, China). Both groups were incubated at 4 °C overnight.

For these assays, the experimental groups were analyzed in biological duplicate; these included the profiling of S9.6-bound DNA in wild-type (WT), LDHA-KO, and lactic acid-treated LDHA-KO cells, alongside LDHA-bound DNA in WT cells. Negative controls, consisting of IgG profiling in WT cells and S9.6 profiling in RNase H-treated WT cells, were performed as single biological replicates.

### 2.4 RNA-sequencing

Total RNA was extracted using TRIzol reagent (Proteinbio, Nanjing, China) according to the manufacturer’s protocol. Transcriptome sequencing was subsequently performed on an Illumina platform by GENEWIZ (Suzhou, China), with three biological replicates analyzed per group. Based on a Wald test, genes exhibiting a |Log_2_(Fold Change)| > 1 and an adjusted *p*-value <0.05 were considered differentially expressed.

Each sample yielded 6 Gb of raw sequencing data, which were analyzed against the mm10 mouse reference genome. Following quality control and filtration, the clean read counts for samples WT1, WT2, WT3, KO1, KO2, and KO3 were 36,085,188, 38,471,046, 44,080,034, 43,076,930, 43,085,136, and 44,742,072, respectively. Sequencing quality was high across all libraries, with Q20 and Q30 scores consistently exceeding 96% and 91%.

### 2.5 Western blot

Cells were treated with 10 mM lactic acid (MedChemExpress, Shanghai, China) or an equivalent volume of vehicle for 24 h. Following treatment, cells were washed with phosphate-buffered saline (PBS) and lysed using radioimmunoprecipitation assay (RIPA) buffer (Thermo Fisher Scientific, Waltham, MA, USA). Protein samples (10 µg per lane) were separated by SDS-polyacrylamide gel electrophoresis (SDS-PAGE). Subsequently, the membranes were blocked with 3% skim milk in Tris-buffered saline with Tween 20 (TBST) for 1 h at room temperature. Membranes were then incubated overnight at 4 °C with the following primary antibodies: anti-Kla (PTM-1401RM, PTMBio, Hangzhou, China), anti-ACTB (AC026, ABclonal, Wuhan, China), anti-γH2AX (AP0687, ABclonal), and anti-LDHA (FNab04735, Finetest, Wuhan, China). After washing, membranes were incubated with HRP-conjugated Goat Anti-Rabbit IgG secondary antibody (AS014, ABclonal) for 1 h at room temperature. Protein bands were visualized using an ECL substrate (Vazyme, Nanjing, China) and imaged with an eBlot Touch Imager (E-Blot, Shanghai, China).

### 2.6 Immunofluorescence microscopy

Cells were treated with lactic acid for 24 h, after which they were harvested and fixed with 4% paraformaldehyde for 20 min at room temperature. Following fixation, cells were permeabilized with 0.1% Triton X-100 in PBS for 10–15 min (standard duration, adjust if different) and then blocked with 10% FBS in PBS for 2 h at room temperature. We added 3 mM magnesium chloride and 1:100 RNase T1 (Yeasen, Shanghai, China) to the blocking buffer to eliminate the interference of double-stranded RNA with the S9.6 antibody, following the guidelines provided by Professor Frederic Chedin (Smolka et al., 2021). After that, cells were incubated overnight at 4 °C with primary antibodies against γ-H2AX (AP0687,ABclonal, Wuhan, China), LDHA (FNab04735, Finetest, Wuhan, China), and S9.6 (65,683, Active Motif, Carlsbad, CA, USA). After three washes with PBS, cells were incubated with an appropriate fluorescently-conjugated secondary antibody (ABclonal) for 2 h at room temperature in the dark. Finally, following three additional PBS washes, slides were mounted using a DAPI-containing mounting medium (YEASEN, Shanghai, China). Images were acquired using a laser scanning confocal microscope (ZEISS, Oberkochen, Germany).

### 2.7 Bioinformatics analysis

Differential genes or enriched peaks were classified based on Gene Ontology (GO) annotations from the GOA database (www.ebi.ac.uk/GOA/) into three primary categories: biological process, cellular component, and molecular function. Biochemical pathways were identified using KEGG database. Subsequently, enrichment analysis was performed using a specific bioinformatic platform (Zhang et al., 2024).

### 2.8 *In vitro* constitution of R-loops by annealing

Two complementary single-stranded DNA sequences and a short complementary single-stranded RNA were synthesized by GENEWIZ (Suzhou, China). Equimolar amounts of the nucleic acids were mixed in T4 PNK buffer (Vazyme, Nanjing, China) to a final concentration of 10 μM. The mixture was then incubated at 95 °C for 10 min, followed by slow cooling to room temperature overnight to allow for annealing. The presence of the synthetic R-loop was confirmed by two methods: immunoprecipitation with the S9.6 antibody and a digestion assay demonstrating its sensitivity to RNase H.

Synthetic DNA sequence (5′ to 3′):

*Csf2ra*-F: CAC​ACA​ATT​TTT​ATT​ATT​TAC​AGT​AAG​CCT​TAA​ACA​GCA​CTC​TGA​CAG​CTG​GGC​AGA​TAT​CTA​CCT​TCT​ATG​TAA​TTA​GAA​TTT​CCT​TTT​TTC​TTT​TCT​TTT​ATT​TTT​TTT​AAT​TCT​CAG​GAC​CTG​CAT​G;

*Csf2ra*-R: CAT​GCA​GGT​CCT​GAG​AAT​TAA​AAA​AAA​TAA​AAG​AAA​AGA​AAA​AAG​GAA​ATT​CTA​ATT​ACA​TAG​AAG​GTA​GAT​ATC​TGC​CCA​GCT​GTC​AGA​GTG​CTG​TTT​AAG​GCT​TAC​TGT​AAA​TAA​TAA​AAA​TTG​TGT​G;

*Znf438*-F: AGG​GGC​ACC​TTT​CCC​ATG​GTC​CTT​TGC​GCT​GTC​TCA​CTT​CTG​CCC​GGA​TAA​AGC​CAC​TCC​CGC​CCG​CCC​GCG​CCC​GGC​CAG​ACA​CGC​CCA​CGA​AGA​GTA​GAC​CAC​GCC​CTT​CCA​GGA​CAA​CCA​CTG​AAA​G;

*Znf438*-R: CTT​TCA​GTG​GTT​GTC​CTG​GAA​GGG​CGT​GGT​CTA​CTC​TTC​GTG​GGC​GTG​TCT​GGC​CGG​GCG​CGG​GCG​GGC​GGG​AGT​GGC​TTT​ATC​CGG​GCA​GAA​GTG​AGA​CAG​CGC​AAA​GGA​CCA​TGG​GAA​AGG​TGC​CCC​T; Synthetic RNA sequence (5′ to 3′):

*Csf2ra:* TTA​TTT​TTT​TTA​ATT​CTC​AGG​ACC​TGC​ATG;

*Znf438*: GGC​ACC​TTT​CCC​ATG​GTC​CTT​TGC​GCT​GTC.

### 2.9 Immunoprecipitation

The synthesized R-loop (10 μL) were combined with S9.6 antibody (2 μg; Abcam, Boston, USA), and the volume was adjusted to 50 μL with nuclease-free PBST. The mixture was incubated for 2 h at 4 °C with rotation to form immunocomplexes. These immunocomplexes were then added to 50 μL of ChIP-grade Protein A/G magnetic beads (ABclonal, Wuhan, China), which had been pre-washed three times with PBST. The slurry was incubated for an additional 2 h at 4 °C with rotation to allow for capture. Following this, the beads were washed three times with PBST on a magnetic rack. Finally, bound material was eluted from the beads by incubation with 20 μL of denaturing elution buffer (Beyotime, Shanghai, China) for 20 min at room temperature. The eluates were then analyzed by PAGE electrophoresis.

### 2.10 Filter DNA binding assay

To evaluate the binding between LDHA and R-loops, a reaction was prepared by combining 2 μg of His-tagged LDHA (ABclonal, Wuhan, China) with 20 μL of synthesized R-loop in 1 mL of buffer A (20 mM Tris-HCl, pH 7.5, 50 mM NaCl, 1 mM EDTA, 2 mM β-mercaptoethanol, 5% glycerol, 0.05 mg/mL BSA) (Fiume et al., 2013). The mixture was incubated at room temperature for 10 min. Following incubation, the mixture was transferred to a centrifugal ultrafiltration device (Merk, Darmstadt, Germany) and centrifuged at 4000 rpm for 10 min to separate protein-R-loop complexes from unbound components. The retentate was then washed three times with 900 μL of buffer A, with each wash followed by centrifugation at 4000 rpm for 10 min. The final, washed retentate was collected for analysis by PAGE electrophoresis.

## 3 Results

### 3.1 Fundamental characteristics of R-loop peaks in 4T1 cells

**FIGURE 1:**
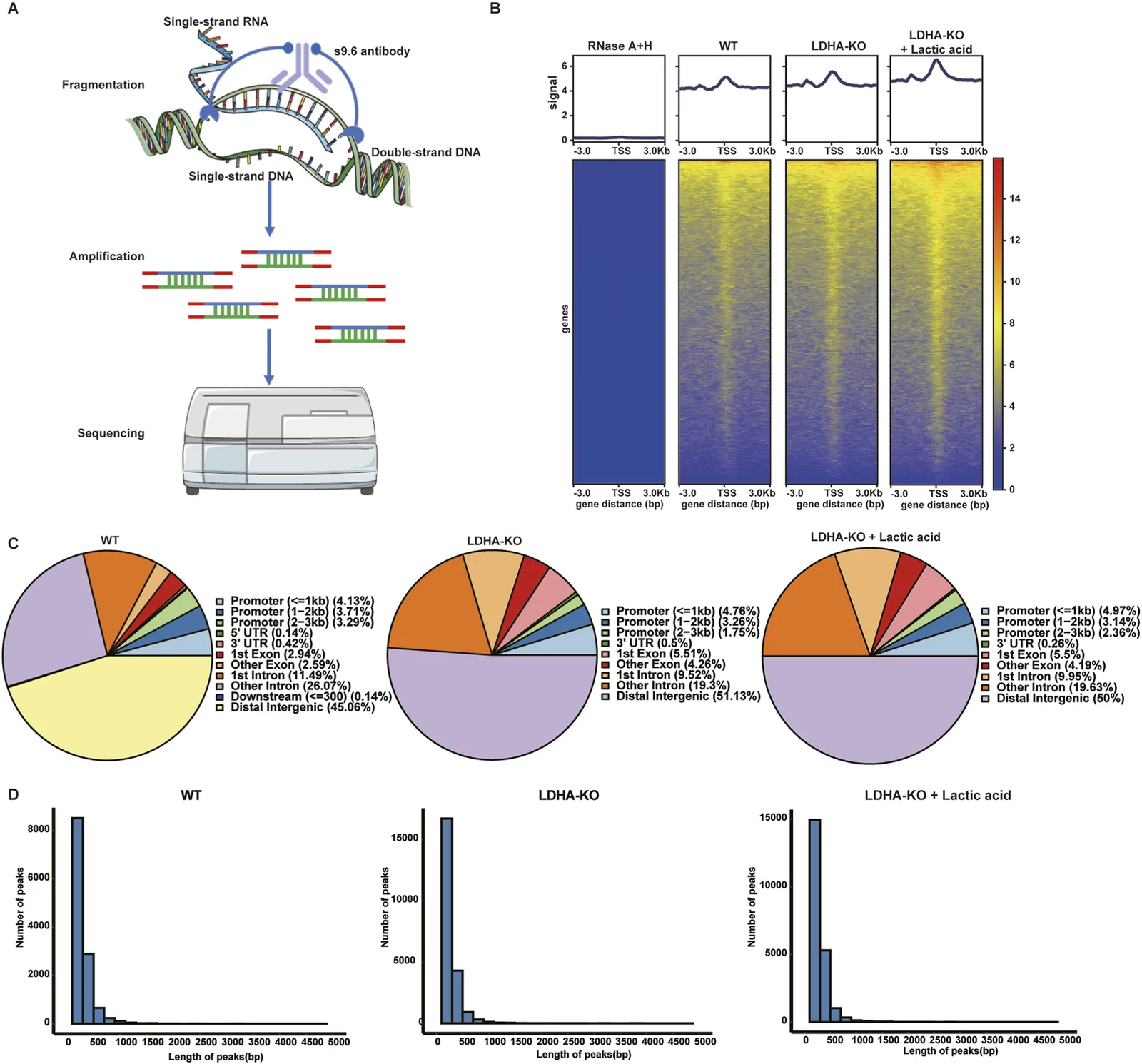
Fundamental characteristics of R-LOOP peaks in 4T1 Cells. **(A)** The experimental process of detecting R-loops through CUT&Tag-seq; **(B)** The relative positions of the s9.6 signals and TSS; **(C)** Gene functional elements corresponding to R-loop enriched peaks; **(D)** The length and numbers of R-loop peaks.

S9.6 antibodies can specifically bind to DNA-RNA hybrids and recognize R-loop sites. We performed CUT&Tag-seq using the S9.6 antibody to map the genomic distribution and abundance of R-loops in wild-type (WT) 4T1 cells, LDHA-knockout (KO) 4T1 cells, and LDHA-KO 4T1 cells supplemented with exogenous lactate, the metabolic product of LDHA (Figure 1A). After obtaining information of R-loops, we first compared their relationship with transcriptional start sites (TSS). As depicted in Figure 1B, pretreatment of the blank group with RNase H resulted in a markedly reduced S9.6 binding signal, confirming good antibody specificity. Data from the experimental groups demonstrated that while R-loop peaks were relatively diffuse, signals were notably stronger near the transcriptional start sites (TSS) and approximately 1.5 kb upstream of the TSS. LDHA knockout or supplementation with exogenous lactate did not cause significant changes in their relative positions.

Next, we analyzed the gene functional elements corresponding to R-loop enriched peaks. In WT cells, approximately 11.13% of R-loop peaks were in promoter regions, 2.94% corresponded to the first exon, 2.59% corresponded to other exons, 11.49% corresponded to the first intron, 26.07% corresponded to other introns, and 45.06% corresponded to distal intergenic regions. Only 0.14% and 0.42% corresponded to the 5′UTR and 3′UTR regions, respectively. Following LDHA knockout, 5′UTR peaks were no longer detected, and the proportions of R-loops across exons, introns, and distal intergenic regions were altered. Furthermore, lactic acid supplementation did not substantially affect the distribution of R-loops in LDHA-KO cells (Figure 1C). Finally, we statistically analyzed the length and number of peaks in each group. As shown in Figure 1D, the length of R-loop peaks was concentrated below 250 bp.

### 3.2 Differential R-loop peaks caused by LDHA knockout

**FIGURE 2:**
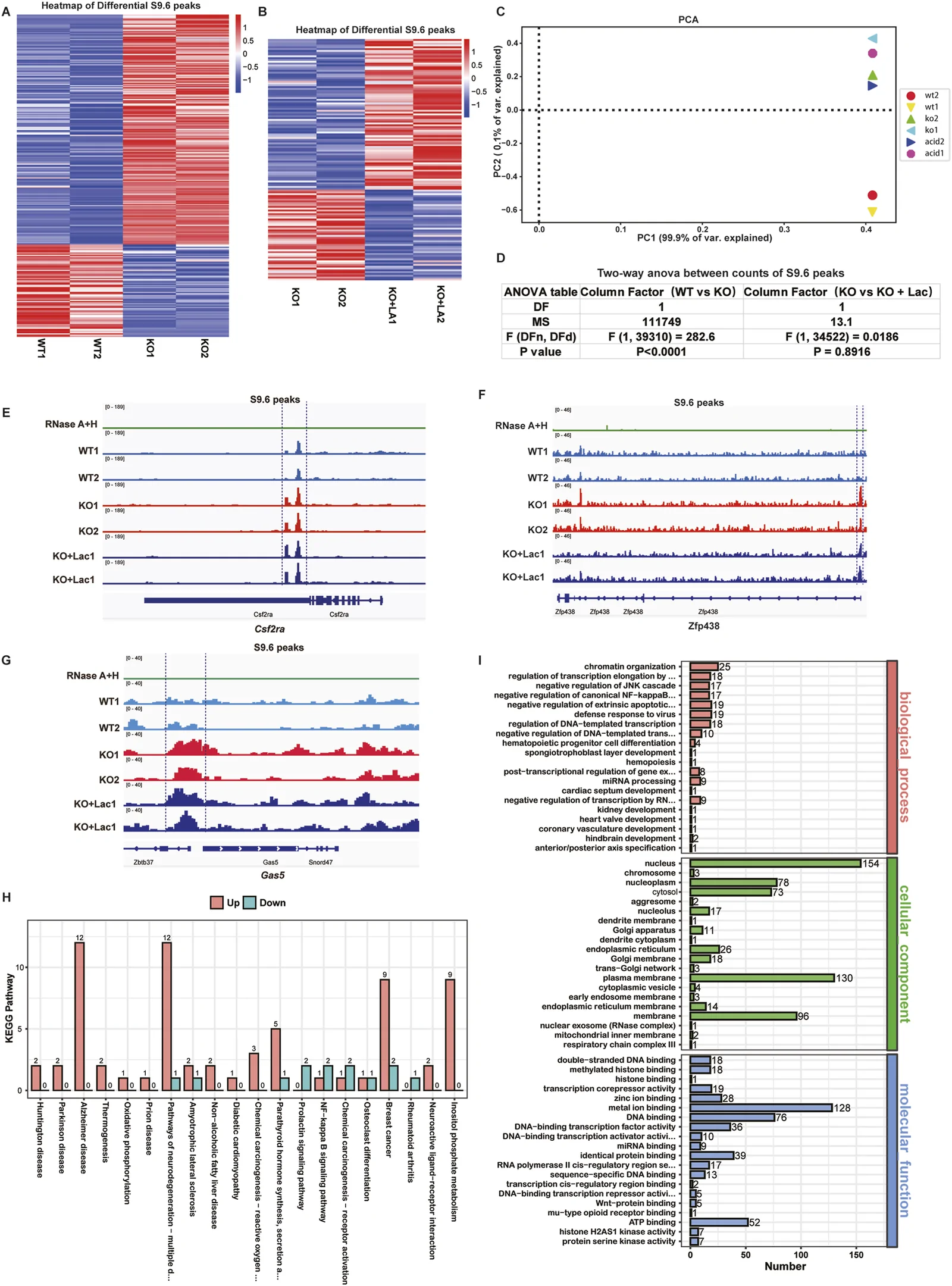
LDHA knockout caused differential R-loop peaks. **(A)** Heatmaps of differential R-loop peaks between WT and LDHA-KO groups; **(B)** Heatmaps of differential R-loop peaks between LDHA-KO and LDHA-KO plus lactic acid groups; **(C)** PCA on S9.6 peaks; **(D)** Two-way Anova analysis on counts of S9.6 peaks; E-G. IGV plots of R-loop peaks on **(E)** *Csf2ra*, **(F)** *Zfp438* and **(G)** *Gas5*; **(H)** KEGG enrichment analysis of the differential peaks; **(I)** GO enrichment analysis of the differential peaks.

We compared the R-loop enrichment peaks in 4T1 cells before and after LDHA knockout. A total of 199 peaks showed a significant increase in count, while 81 peaks showed a significant decrease in count (Figure 2A; Supplementary Table S1). When mapped to genes, 189 genes exhibited upregulated R-loop peaks, and 76 genes showed downregulated R-loop peaks. In the LDHA-KO cells, after exogenous lactic acid supplementation, 83 peaks showed increased counts, while 50 peaks showed decreased counts (Figure 2B; Supplementary Table S2). Mapped to genes, 80 genes had upregulated peaks, and 50 genes had downregulated peaks. To determine whether LDHA knockout broadly affects R-loop enrichment, we performed a principal component analysis (PCA) and a two-way analysis of variance (ANOVA) (Figures 2C,D). This revealed that LDHA knockout significantly altered R-loop peak counts (two-way ANOVA, p < 0.0001). Given that LDHA is a metabolic enzyme, we hypothesized that its catalytic activity might mediate this regulation. To test this, we supplemented LDHA-knockout cells with exogenous lactate, the metabolic product of LDHA; however, this intervention did not significantly alter R-loop abundance (two-way ANOVA, p = 0.8916).

To more intuitively reflect the impact of LDHA knockout on R-loop enrichment peaks, we selected three representative loci for visualization using IGV, including *Csf2ra, Zfp438 and Gas5* (Figures 2E–G). CSF2RA, an essential subunit of the GM-CSF receptor, is highly expressed in breast cancer and represents a potential immune-related biomarker. ZFP438 is a zinc-finger protein predicted to possess sequence-specific, RNA polymerase II-associated DNA-binding transcription factor activity and to participate in the negative regulation of DNA-templated transcription. GAS5 encodes a long noncoding RNA with tumor-suppressive functions in the regulation of apoptosis and autophagy-related metabolic pathways. GAS5 is downregulated in breast cancer and is associated with poor prognosis and reduced overall survival. Following LDHA knockout, *Csf2ra* on chromosome 19 at positions 61223879-61224102 and 61266384-61267254 showed a 2.2-fold and 1.55-fold increase in peak counts, respectively (Figure 2E). *Zfp438* on chromosome 18 at position 5334428-5334926 showed a 3.85-fold increase in peak counts (Figure 2F). *Gas5* on chromosome one at position 161034564-5 161035071 showed a 1.71-fold increase in peak counts (Figure 2G). In contrast, exogenous lactate supplementation did not significantly alter R-loop enrichment at these loci.

Finally, we performed pathway enrichment analysis on the differential peaks caused by LDHA knockout. The Kyoto Encyclopedia of Genes and Genomes (KEGG) enrichment analysis (Figure 2H) revealed that the differential peaks were mainly enriched in the pathways of Alzheimer’s disease, neurodegenerative diseases, and breast cancer. Gene Ontology (GO) enrichment analysis showed (Figure 2I) that the biological process most affected by the differential peaks was chromatin organization, with the largest affected cellular component being the nucleus, and the most impacted molecular function being metal ion binding. In conclusion, LDHA knockout altered genome-wide R-loop enrichment, and this effect was not reversed by exogenous lactate supplementation. We therefore propose that LDHA modulates R-loop dynamics through physical interactions.

### 3.3 The DNA sequences bound by LDHA partially overlap with the R-loop region

**FIGURE 3:**
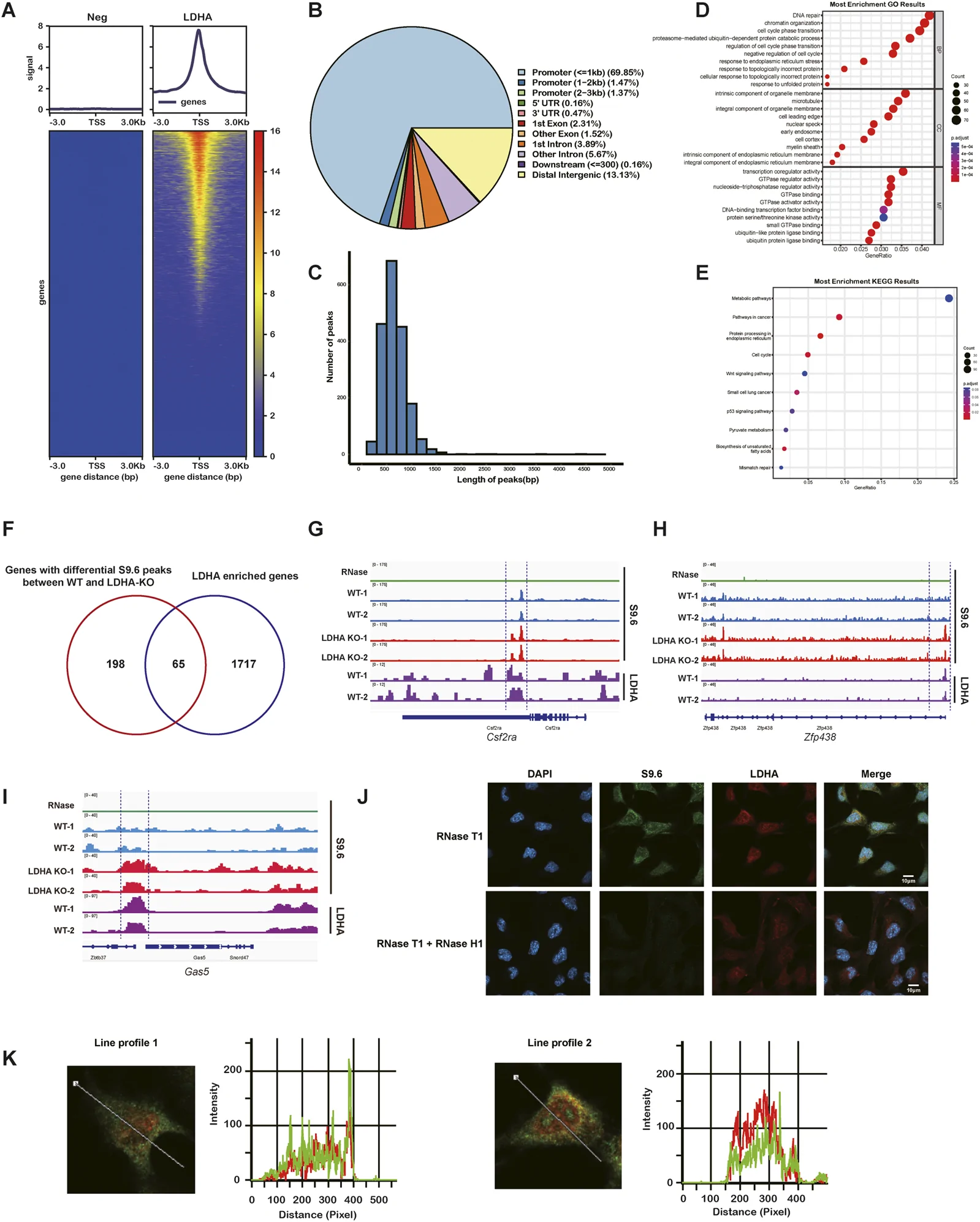
LDHA bound to parts of the R-loop enrichment regions. **(A)** The relative positions of LDHA binding sites and TSS; **(B)** Gene functional elements corresponding to LDHA enriched peaks; **(C)** The length and numbers of LDHA-enriched peaks. **(D)** GO enrichment analysis of LDHA-enriched peaks; **(E)** KEGG enrichment analysis of LDHA-enriched peaks; **(F)** Venn diagram of LDHA-enriched genes and differential R-loop peaks; G-I. IGV plots of LDHA-enriched peaks and R-loop peaks on **(G)** *Csf2ra*, **(H)** *Zfp438* and **(I)** *Gas5*; **(J,K)** Immunofluorescence staining revealed the co-localization of S9.6 and LDHA, line profiles were conducted with Image-Pro.

LDHA is a single-stranded DNA-binding protein. To investigate whether LDHA influences R-loops by direct binding to genomic DNA, we used CUT&Tag-seq to identify its binding sites. First, we performed a basic analysis of the LDHA enrichment peaks. As shown in Figure 3A, LDHA binding sites are concentrated near transcription start sites (TSS). The gene functional elements corresponding to these peaks are primarily promoters within 1 kb of the TSS (Figure 3B), and the peak lengths are mainly between 250 and 1000 bp (Figure 3C). GO enrichment analysis revealed that the biological process most affected by LDHA-enriched peaks is DNA repair, the cellular component most affected is the intrinsic component of organelle membranes, and the molecular function most affected is transcription coregulator activity (Figure 3D). KEGG pathway enrichment analysis indicated that LDHA-enriched peaks are concentrated in metabolic pathways and tumor-related pathways (Figure 3E).

Next, we performed a joint analysis of LDHA-enriched peaks and R-loop-enriched peaks. As shown in Figure 3F, after LDHA knockout, R-loop-enriched peaks on 263 genes showed significant changes, among which 65 genes had LDHA binding sites. Representative IGV plots were shown for three genes, where LDHA precisely binds to the regions corresponding to the differential R-loop peaks of *Csf2ra* (Figure 3G), *Zfp438* (Figure 3H), and *Gas5* (Figure 3I). Furthermore, we employed immunofluorescence to examine the cellular distribution of LDHA and R-loops. Given previous reports that the S9.6 antibody may cross-react with double-stranded RNA during immunofluorescence, we treated the slides with RNase T1 to mitigate non-specific binding. As a control, parallel samples were treated with RNase H1 and RNase T1. This treatment significantly diminished the S9.6 fluorescence signal (Figure 3J), confirming the specificity of the S9.6 antibody for R-loops under these conditions. Figure 3K revealed that both LDHA and R-loops were distributed throughout the nucleus and cytoplasm, and the line profile analysis demonstrated their partial colocalization.

Finally, detailed information on LDHA enrichment peaks is provided in Supplementary Table S3.

Therefore, we propose that LDHA bind to partial R-loop regions and influence their homeostasis.

### 3.4 LDHA bound with synthesized R-loop-like substrates *in vitro*

**FIGURE 4:**
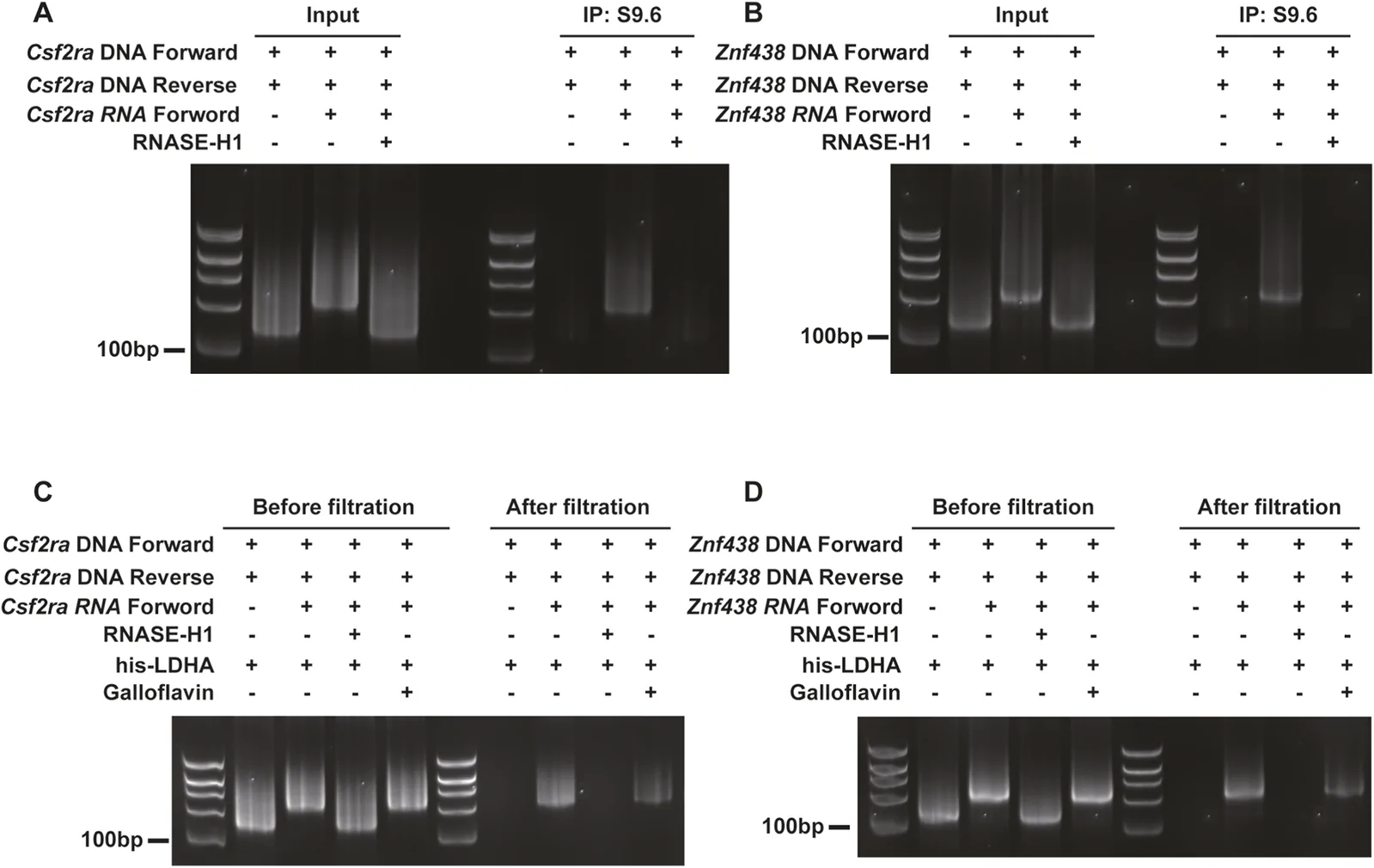
LDHA bound to R-loop *in vitro*. Immunoprecipitation of synthetic R-loop structures from the **(A)** *Csf2ra* and **(B)** *Znf438* loci was performed using the S9.6 antibody, and the successful pulldown was confirmed by gel electrophoresis. The interaction between LDHA and synthetic R-loop structures from the **(C)** *Csf2ra* and **(D)** *Znf438* loci was analyzed by a filter DNA-binding assay, and the binding was subsequently visualized by PAGE electrophoresis.

We synthesized two complementary single-stranded DNA sequences and a short complementary single-stranded RNA sequence *in vitro*, which were annealed using a heat-based method to artificially form the RNA:DNA hybrid-containing R-loop-like structures. As demonstrated in Figures 4A,B, the S9.6 antibody exhibited strong binding to the generated R-loop-like structures, whereas its binding to double-stranded DNA was substantially weaker. Furthermore, following RNase H treatment, the binding of the S9.6 antibody to the R-loop was reduced. These results confirmed the successful *in vitro* synthesis of R-loop-like structures.

A Filter DNA-binding assay was employed to investigate the *in vitro* interaction between LDHA and the synthesized R-loop. The results, depicted in Figures 4C,D, demonstrated that LDHA selectively bound to the R-loop, causing its retention in the ultrafiltration tube, whereas double-stranded DNA was not retained. Notably, this interaction was abolished by either treatment with RNase H, which degrades the R-loop’s RNA strand, or the addition of Galloflavin, which disrupts LDHA’s binding to single-stranded DNA.

Thus, LDHA was demonstrated to interact with the R-loop-like structures. However, this assay cannot determine whether LDHA specifically recognizes the RNA:DNA hybrid, the displaced single-stranded DNA, or another structural feature.

### 3.5 Transcriptome alterations induced by LDHA and their association with R-loops

**FIGURE 5:**
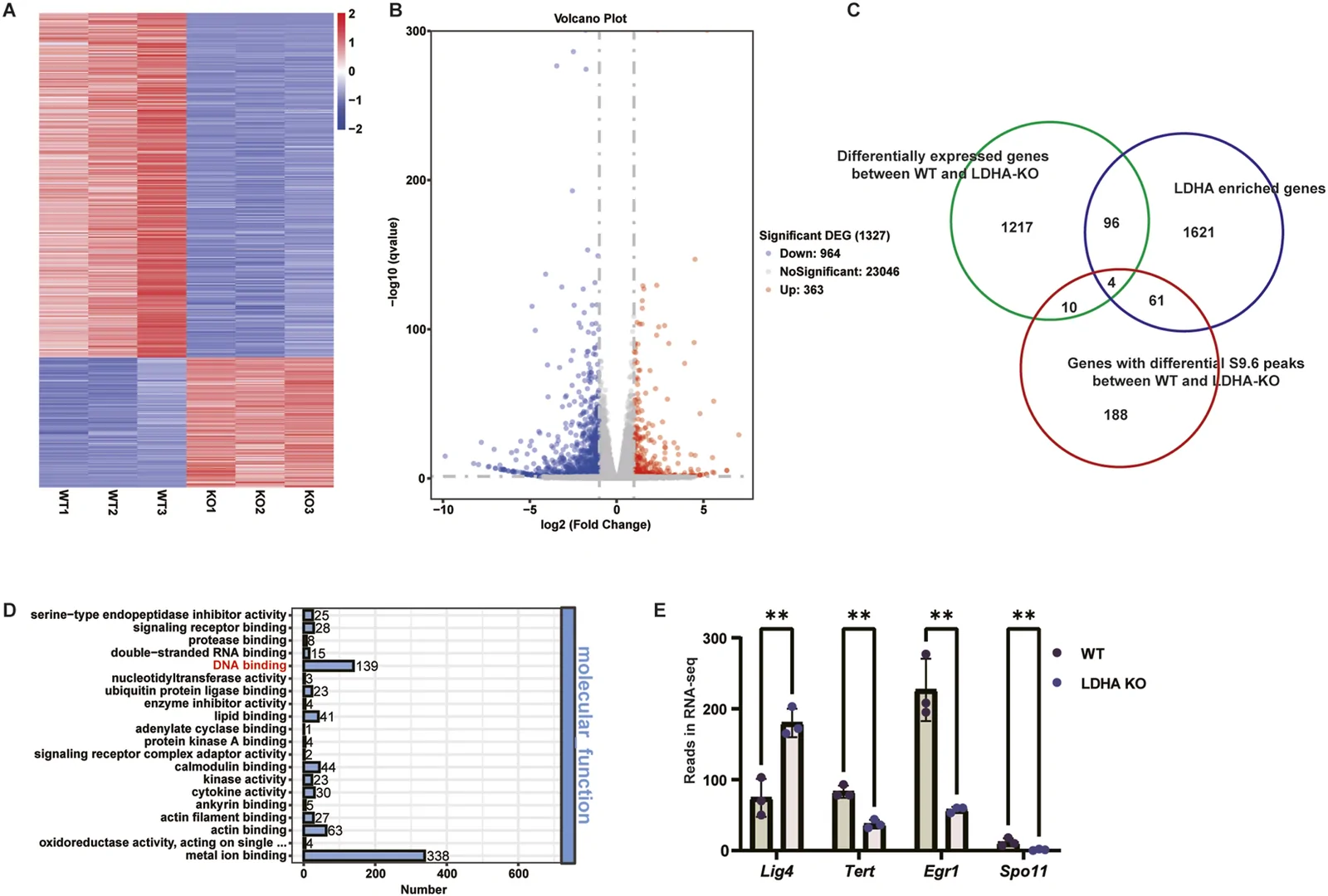
Transcriptome alterations induced by LDHA depletion and their association with R-loops. **(A)** Heatmap and **(B)** volcano plot of differentially expressed genes after LDHA knockout in 4T1 cells; **(C)** Venn diagram of LDHA-enriched genes, differential R-loop peaks and differential expressed genes; **(D)** GO enrichment of molecular function on differential expressed genes; **(E)** mRNA expression of Lig4, Tert, Egr1 and Spo11 in WT and LDHA-KO 4T1 cells (n = 3, two-tailed Student’s t-test, **p < 0.01).

R-loops play a crucial role in regulating transcription. To further investigate the relationship between LDHA and R-loops, we performed transcriptome sequencing analyses before and after LDHA knockout. As illustrated in Figures 5A,B and Supplementary Tables S4, S363 genes were significantly upregulated, while 964 genes were significantly downregulated. However, integrating the differentially expressed genes with R-loop differential peaks and LDHA binding sites (Figure 5C) revealed that only 14 out of the 1,327 differentially expressed genes were potentially influenced by R-loops, and merely four genes exhibited both R-loop differential peaks and LDHA binding sites simultaneously. While these findings do not support the hypothesis that LDHA regulates gene transcription through R-loop binding, a potential role for R-loops in LDHA-mediated transcriptional regulation cannot be entirely ruled out, given their transient and site-specific nature.

Interestingly, 198 R-loop differential peaks were identified without LDHA binding (Figure 5C), raising the possibility that LDHA might indirectly influence R-loop stability through its regulatory effects on transcription. To explore this hypothesis, we conducted Gene Ontology (GO) enrichment analysis on the affected molecular functions. As shown in Figure 5D, 139 DNA-binding-related genes exhibited significant changes in expression, suggesting that these DNA-binding proteins may contribute to R-loop homeostasis. For example, during replication fork stalling caused by R-loops, Lig4 forms a complex with XRCC4 to reconnect DNA ends cleaved by MUS81, thereby facilitating replication fork restart (Rao et al., 2024; Chappidi et al., 2020). Similarly, Tert forms a reverse transcriptase-like RNA-DNA hybrid structure by binding RNA templates and telomeric DNA (Qi et al., 2012), and R-loop accumulation increases when Tert expression decreases (Catto et al., 2023). Additionally, Egr1’s zinc finger structure recognizes DNA conformations highly similar to RNA-DNA hybrids (Marco et al., 2002), and Spo11 induces programmed DNA double-strand breaks (DSBs) during meiosis, with hotspot formation potentially linked to R-loops (Liu et al., 2023). As shown in Figure 5E, the transcriptional profiles of these R-loop-related genes changed significantly following LDHA knockout: Lig4 expression increased, while *Tert*, *Egr1*, and *Spo11* expression significantly decreased.

### 3.6 Increased R-loop accumulation and genomic instability following LDHA knockout

**FIGURE 6:**
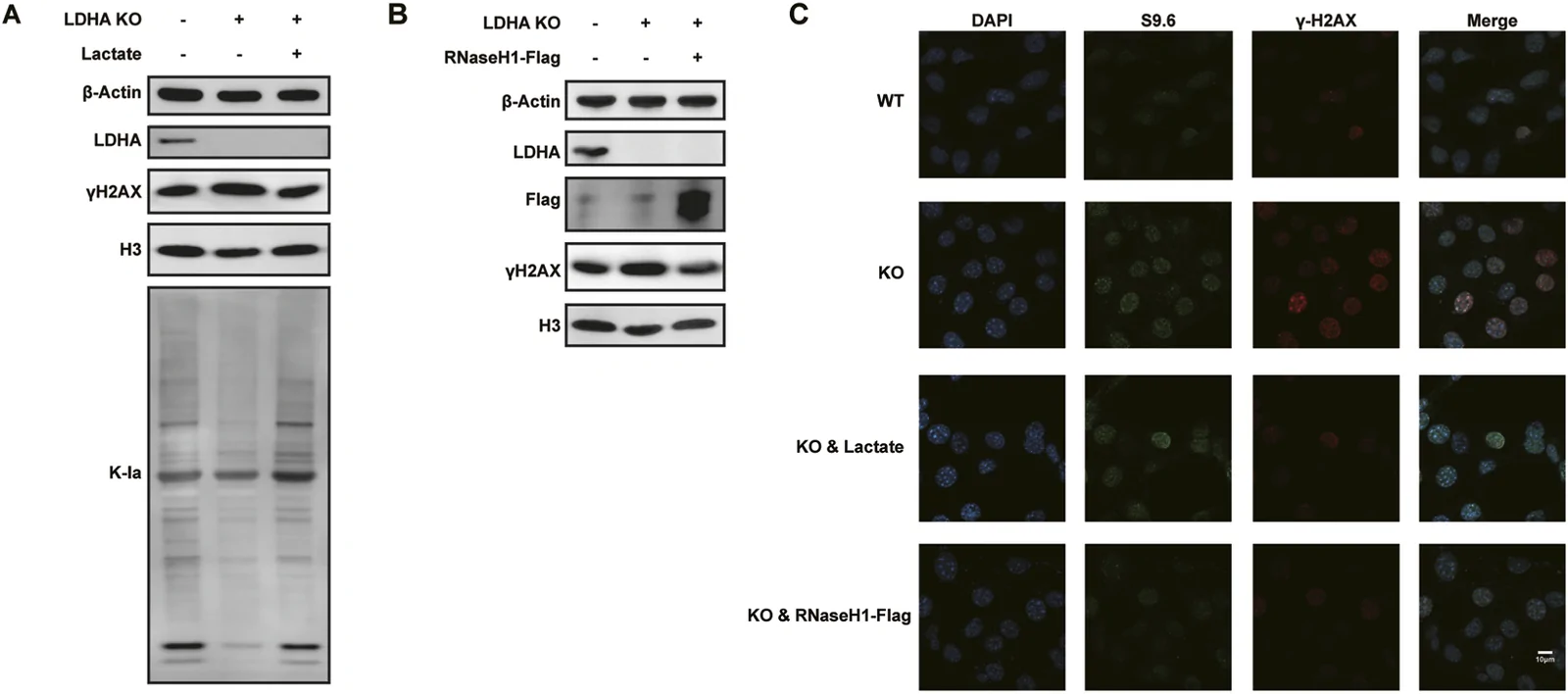
LDHA knockout induces R-loop accumulation and genomic instability. **(A,B)** Immunoblot analysis of Kla, γ-H2AX, H3, Flag, and LDHA expression in the indicated cell lines. **(C)** Immunofluorescence imaging showing the distribution and intensity of γ-H2AX and S9.6 signals in the indicated cell lines.

R-loops play essential physiological roles in DNA repair processes, but their aberrant accumulation or persistence is a major contributor to genomic instability. To investigate the role of LDHA in DNA damage, we examined the effects of LDHA knockout. As illustrated in Figure 6A, LDHA knockout led to an elevated expression of the DNA damage marker γ-H2AX, while exogenous lactate supplementation partially reduced γ-H2AX levels. We next explored whether R-loops participated in the DNA damage induced by LDHA knockout. Overexpression of RNase H1-Flag in LDHA-KO cells attenuated γ-H2AX levels (Figures 6B,C) and diminished the nuclear accumulation of R-loops (Figure 6C). These results indicate that R-loops contribute to the genomic instability caused by LDHA knockout.

## 4 Discussion

LDHA is a key metabolic enzyme that plays a critical role in the progression of kidney injury, Alzheimer’s disease, hyperoxaluria, and various tumors (Azushima et al., 2023; Tian et al., 2023; Sharma et al., 2022; Syed, 2023). Notably, Nedosiran, a drug targeting LDHA for the treatment of hyperoxaluria, was approved by the FDA in September 2023 (Syed, 2023). This highlights the importance of further elucidating the biological functions of LDHA. Previous studies have identified LDHA as a single-stranded DNA-binding protein (Grosse et al., 1986), leading us to hypothesize that LDHA may influence R-loop stability. In our study, LDHA knockout in 4T1 cells resulted in significant changes in R-loop peak signals (*p* < 0.0001). Although lactate, a metabolic product of LDHA, was hypothesized to mediate LDHA’s effects on R-loops through lactylation modifications of regulatory proteins, our experiments revealed that exogenous lactate supplementation caused only minor changes in R-loop peak signals and did not significantly alter the overall R-loop landscape (P = 0.8916). These findings indicate that LDHA affects the R-loop landscape independently of its metabolic product, lactate. Furthermore, we demonstrated that LDHA binds to R-loop-like structures. CUT&Tag-seq analysis of LDHA-bound regions revealed partial overlap between LDHA-binding sites and differential R-loop peaks (65/263), suggesting that LDHA may influence R-loop homeostasis through direct binding.

R-loops are widely present in actively transcribed genomic regions and regulate gene expression and chromatin structure (Petermann et al., 2022). Consequently, we evaluated the impact of LDHA on transcription and its association with R-loops. LDHA knockout significantly affected gene transcription, leading to the upregulation of 363 genes and downregulation of 964 genes. However, only four differentially expressed genes overlapped with both differential R-loop peaks and LDHA binding peaks. These findings do not support the hypothesis that LDHA regulates gene transcription through R-loop binding; rather, mechanisms such as histone lactylation and reactive oxygen species (ROS) are more likely to mediate LDHA’s transcriptional effects. Interestingly, LDHA knockout influenced the transcription of several DNA-binding proteins, including Lig4, Tert, Egr1, and Spo11. These altered DNA-binding proteins might contribute to LDHA’s effects on R-loops.

R-loops can exacerbate transcription-replication conflicts, impair DNA replication fork progression, and mediate genomic instability (Camino et al., 2023; Hamperl et al., 2017). Conversely, in Arabidopsis chloroplasts, R-loops have been shown to positively promote the activity of WHY1/3 and RecA1, thereby ensuring homologous recombination repair and organelle development (Wang et al., 2021). In human cells, BRCA1 is recruited to R-loops formed at some transcription termination sites, where the BRCA1/SETX complex supports a repair mechanism to resolve DNA damage caused by R-loops at transcriptional pause sites (Hatchi et al., 2015). In our study, LDHA knockout resulted in increased R-loop foci formation and elevated levels of the DNA damage marker γ-H2AX. Exogenous lactate supplementation and RNase H1 overexpression both partially restored the genomic stability of 4T1 cells. R-loops therefore contribute to LDHA knockout-associated DNA damage; however, the precise mechanisms warrant further investigation.

## 5 Conclusion

In this study, we identify a novel association between LDHA and R-loops. We establish that LDHA modulates R-loop stability, thereby safeguarding genomic stability. Collectively, these findings deepen our understanding of the non-canonical functions of LDHA and provide novel experimental evidence that expands the framework of its pleiotropic biological roles.

## 6 Limitations

The current study did not elucidate the detailed molecular mechanisms through which LDHA regulates R-loops, such as the involvement of specific molecular chaperones or protein-protein interactions. Additionally, the precise pathways by which LDHA influences genomic stability *via* R-loop modulation warrant further investigation. Finally, as our experiments were conducted exclusively in murine breast cancer cell line, further validation across diverse species, including human cells, is necessary to confirm the generalizability of these findings.

## Data Availability

The datasets presented in this study can be found in online repositories. The names of the repository/repositories and accession number(s) can be found below: https://www.ncbi.nlm.nih.gov/, PRJNA1288022.
