# Supplementary material for: Loss of lactate dehydrogenase A disrupts R-loop homeostasis and induces DNA damage in breast cancer cells
Source: Front Genet. 2026 Sep 14;17:1942597. doi: 10.3389/fgene.2026.1942597 (PMC13616447; doi:10.3389/fgene.2026.1942597)
Supplement: Supplementary file 1 [file DataSheet2.pdf]

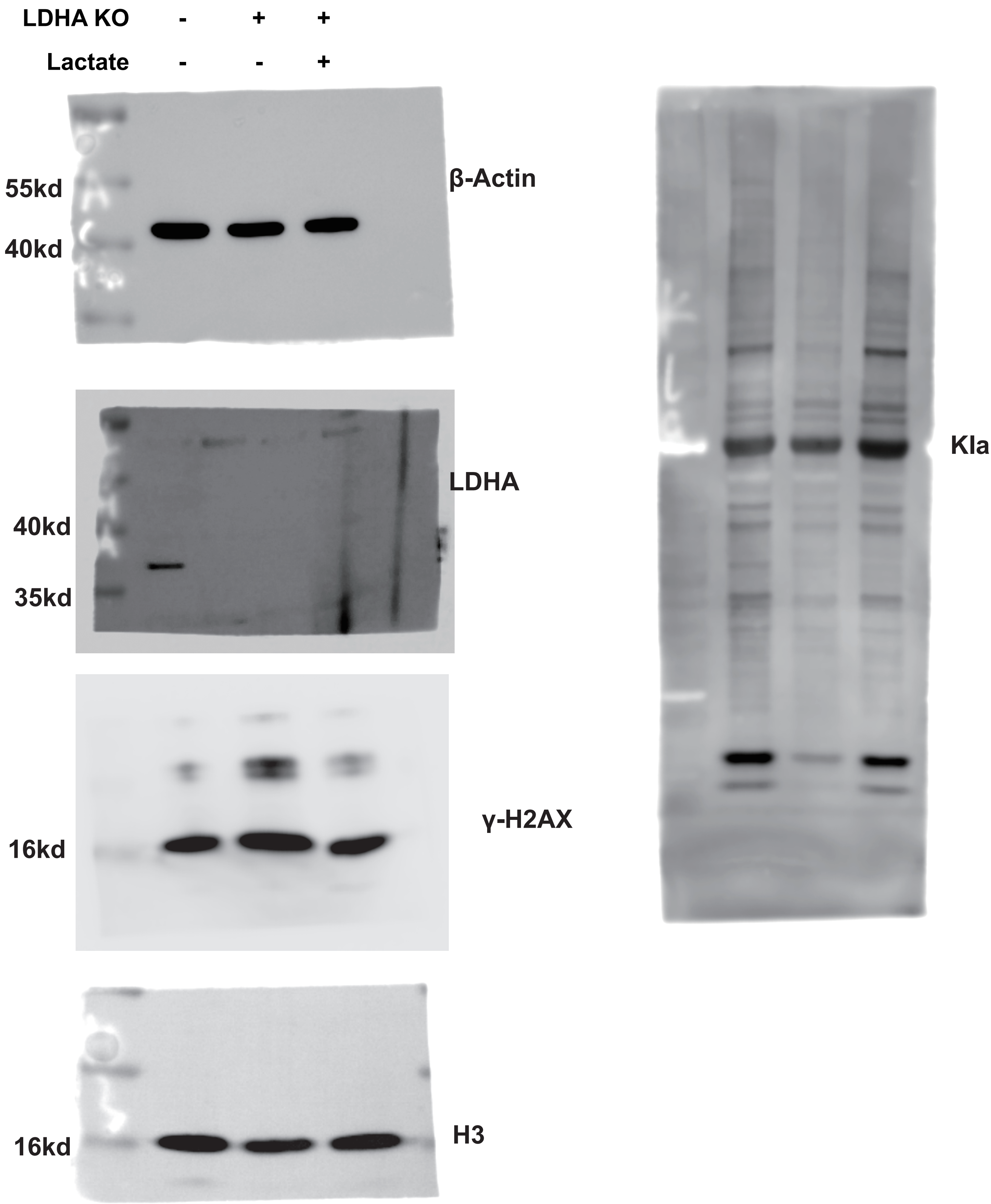

LDHA KO - + +
Lactate - - +
55kd
40kd
β-Actin
LDHA
40kd
35kd
γ-H2AX
16kd
16kd
H3
Kla

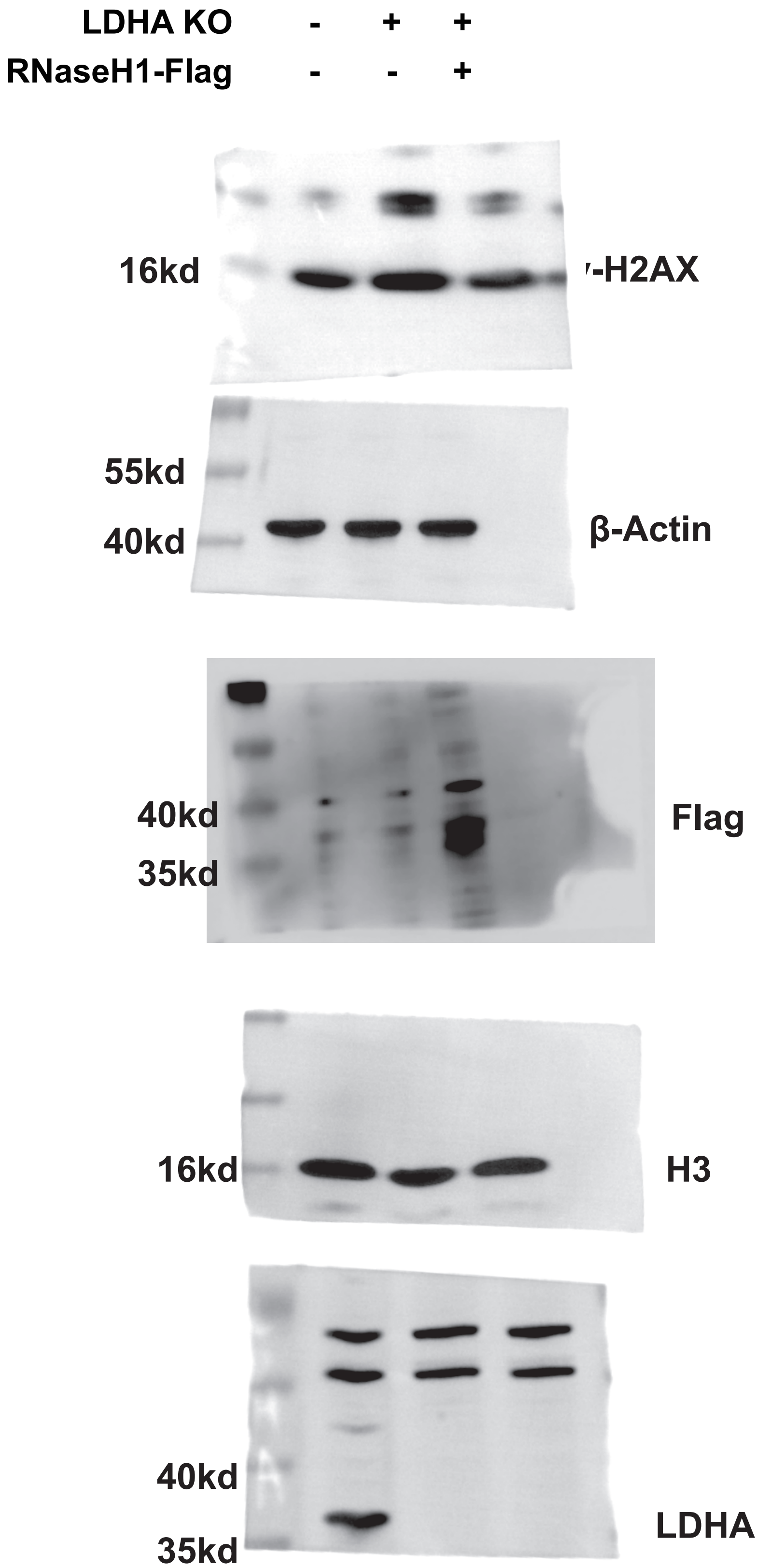
LDHA KO - + +
RNaseH1-Flag - - +
16kd
-H2AX
55kd
40kd
β-Actin
40kd
35kd
Flag
16kd
H3
40kd
35kd
LDHA
